# Infection of *C. elegans* by *Haptoglossa* Species Reveals Shared Features in the Host Response to Oomycete Detection

**DOI:** 10.3389/fcimb.2021.733094

**Published:** 2021-10-14

**Authors:** Manish Grover, Michael K. Fasseas, Clara Essmann, Kenneth Liu, Christian Braendle, Marie-Anne Félix, Sally L. Glockling, Michalis Barkoulas

**Affiliations:** ^1^ Department of Life Sciences, Imperial College, London, United Kingdom; ^2^ Université Côte d’Azur, CNRS, Inserm, IBV, Nice, France; ^3^ Institut de Biologie de l’Ecole Normale Supérieure, CNRS, Inserm, Paris, France; ^4^ Independent Researcher, Eastbourne, United Kingdom

**Keywords:** *chitinase-like*, CLPs, gun cells, haptoglossa, oomycete, innate immunity

## Abstract

Oomycetes are a group of eukaryotic organisms that includes many important pathogens of animals and plants. Within this group, the *Haptoglossa* genus is characterised by the presence of specialised gun cells carrying a harpoon-like infection apparatus. While several *Haptoglossa* pathogens have been morphologically described, there are currently no host systems developed to study the infection process or host responses in the lab. In this study, we report that *Haptoglossa* species are potent natural pathogens of *Caenorhabditis* nematodes. Using electron microscopy, we characterise the infection process in *C. elegans* and demonstrate that the oomycete causes excessive tissue degradation upon entry in the body cavity, whilst leaving the host cuticle intact. We also report that the host transcriptional response to *Haptoglossa* infection shares similarities with the response against the oomycete *Myzocytiopsis humicola*, a key example of which is the induction of *chitinase-like (chil)* genes in the hypodermis. We demonstrate that this shared feature of the host response can be mounted by pathogen detection without any infection, as previously shown for *M. humicola*. These results highlight similarities in the nematode immune response to natural infection by phylogenetically distinct oomycetes.

## Introduction

Studying biological organisms in their natural context can reveal specific features that organisms have evolved for survival in their ecological habitat. Such features may include specific anatomical and molecular responses to natural pathogens that occupy the same niche and pose an infection threat. For example, in *C. elegans*, the swollen-tail phenotype is observed in response to bacterial infection by *Microbacterium nematophilum* (Hodgkin et al., 2000); secretion of antimicrobial peptides of the *nlp (neuropeptide-like proteins)* and *cnc (caenacin)* families characterises the response to fungal infection by *Drechmeria coniospora* (Pujol et al., 2008; Zugasti and Ewbank, 2009); and finally, the intracellular pathogen response (IPR) is induced when *C. elegans* is challenged with microsporidia, such as *Nematocida parisii* (Reddy et al., 2017). Therefore, expanding our knowledge on natural pathogens can help us understand the battery and specificity of innate immune responses that are relevant within a complex wild environment.

Oomycetes are filamentous eukaryotic microbes which superficially resemble fungi but are phylogenetically distinct (Beakes et al., 2012). They exist in both terrestrial and aquatic environments and several of them are successful pathogens of plants and animals (Derevnina et al., 2016). Notable examples include *Phytophthora infestans*, which causes late blight of potato (Fry, 2008) and *Pythium insidiosum*, which causes pythiosis in humans and other mammals (De Cock et al., 1987). Due to a paucity of experimentally tractable model systems, animal oomycete infections have been understudied compared to plant infections. We have previously identified an oomycete, *Myzocytiopsis humicola*, as a natural pathogen of *C. elegans* (Osman et al., 2018), which allows to make use of the plethora of tools available for research on this model organism to understand animal-oomycete interactions. We have shown that exposure to *M. humicola* leads to induction of the host *chitinase-like (chil)* genes in the hypodermis, with *chil-27* being a member of this family previously used as a marker of infection (Osman et al., 2018). CHIL proteins are thought to be catalytically inactive chitinases and may act by modifying the cuticle properties, thereby antagonising infection by limiting pathogen attachment to the host (Osman et al., 2018). Interestingly, *chil* gene induction is part of a broader oomycete recognition response (ORR) programme, which is likely to be triggered upon neuronal detection of a pathogen-associated molecular pattern that remains currently unknown (Fasseas et al., 2021).

In this study, we report additional oomycete species belonging to the *Haptoglossa* genus as potent pathogens of *C. elegans*. *Haptoglossa* constitutes the earliest diverging clade within the oomycete lineage along with *Eurychasma* and *Haliphthora* (Beakes et al., 2012). *Haptoglossa* was initially described as an obligate endoparasite of nematodes and rotifers living in soil and decaying vegetation (Drechsler, 1940; Barron, 1980; Robb and Barron, 1982). Since then, multiple species have been investigated at the morphological level (Glockling and Beakes, 2000b; Glockling and Beakes, 2000c; Glockling and Beakes, 2001; Hakariya et al., 2007), but no model host has been developed to systematically study these unusual pathogens. The genus is characterized by the production of unique injection cells, known as gun cells, which physically rupture the cuticle of the host to transfer a single-celled sporidium inside the host to initiate the infection. These gun cells have granted *Haptoglossa* the stature of being nature’s “ballistic missile” (Robb and Barron, 1982). The gun cells show remarkable diversity in their shape and size and arise from flagellated motile zoospores or non-motile aplanospores depending on the species (Glockling and Beakes, 2000a; Hakariya et al., 2007). We investigate the host response to *Haptoglossa* infection and report similarities with what has previously been discovered for *M. humicola* recognition (Fasseas et al., 2021). We finally reveal that distinct *Haptoglossa* strains from different geographical locations are all capable of triggering *chil-27* gene induction, which highlights a common response associated with oomycete recognition in *C. elegans*.

## Materials and Methods

### Strains and Oligos Used in the Study

Most nematode strains including *C. elegans* N2, CB4856, *Caenorhabditis briggsae* AF16, *Caenorhabditis remanei* PB4641, *Caenorhabditis angaria* RGD1, *Oscheius tipulae* CEW1, *Pristionchus pacificus* PS312 were obtained from the Caenorhabditis Genetics Center (CGC), USA. *Mesorhabditis spiculigera* MBA1180 was isolated from rotten lemons in Athens, Greece. *Haptoglossa zoospora* MBAo1 was isolated from a manure pile near Sandy in Bedfordshire, UK. *Haptoglossa sp*. JUo6 was isolated on *Oscheius tipulae* from rotting fruits of *Magnolia hypoleuca* sampled in the Botanical garden of the University of Sendai in Japan. NICo1 was isolated on *Caenorhabditis tropicalis* from rotten figs in Manzhou Township, Pingtung County, Taiwan. The oomycete *Myzocytiopsis humicola* JUo1 and strains JU2519 and MBA281 containing icbIs4[pGO4, pCMH1195] II were described in a previous study (Osman et al., 2018). *C. elegans* strain N2 was cultured on NGM plates seeded with *E. coli* OP50 at 20°C under standard conditions (Stiernagle, 2006). All oomycete isolates were grown and maintained at 25°C on standard NGM plates along with *C. elegans* N2 (Stiernagle, 2006). The cultures can be maintained indefinitely by adding fresh N2 whenever the plate is running out of food in the case of *Haptoglossa*. Molecular characterization of *Haptoglossa* isolates was performed using the following primers for *cox-2* and 18S: Cox-2F:GGCAAATGGGTTTTCAAGATCC and Cox-2R: CCATGATTAATACCACAAA, Hap-18SF : ATTAACTGTGCGGATCGTGC and Hap-18SR : CATAGTACGCACGCACCAAA. FISH was performed as previously described (Osman et al., 2018).

### Pathogen Extract Preparation

Pathogen and control extracts were prepared as previously described (Fasseas et al., 2021). Briefly, 90 mm plates showing advanced stage *Haptoglossa* infection were thoroughly washed with 3 ml H_2_O with simultaneous scrapping of the plate surface to recover as many dead worms as possible. The suspension was centrifuged either at 1000xg or 12,000xg for 5 min and the supernatant was passed through a 0.2μm syringe filter to obtain an extract. Extracts obtained by centrifuging the suspension at 1000xg speed and keeping the supernatant resulted in higher numbers of worms showing *chil-27p::GFP* induction. Extracts from the gun cells and thalli of the aplanosporic *Haptoglossa* strain NICo1 were made by transferring clusters of gun cells or worms containing early-stage thalli into 100 μl H_2_O. The mixtures were heated at 95°C for 20 minutes and allowed to cool before use in phenotypic assays. *M. humicola* extract was prepared by picking sporangia filled animals into 100 µl H_2_O which was then heated at 95°C for 20 minutes. A control extract was made in the same manner using N2 adults into 100 μl H_2_O.

### Phenotypic Assays

All infection assays were performed at 25°C in triplicates on standard NGM plates with a small lawn of *E. coli* OP50 made using 100 µl of bacterial culture. Infection assays were started by moving 30 L4 animals to plates containing 10 infected worms and 3 such plates were used per condition (n=90 animals in total). The animals were scored for visible infection (i.e. development of spherical sporangia for *M. humicola* JUo1 and sausage-like thalli for *Haptoglossa* isolates) and the live animals were moved to new plates without any pathogen every 48 hours until all the animals in the assay were dead. Dead animals without any signs of infection or those that got lost were censored. All experiments were reproduced at least three times and GraphPad Prism 7 (GraphPad Software Inc.) was used to plot and compare survival curves. The log-rank test was used to assess statistical significance and a *p* value of <0.05 was considered significant.

For induction assays, the extracts were made as described above and 100-150 µl was added to OP50 plates to fully cover the bacterial lawn. Around 100 synchronized *C. elegans* L2 stage worms or ~ 400 eggs were added to each plate. The plates were incubated at 20^°^C and scored for *chil-27p::GFP* induction after 24h or 48h respectively using a Zeiss Axio Zoom V16 (Zeiss) microscope. Animals were classified in two groups (GFP positive and negative) and *col-12p::mCherry* expression was confirmed in both cases. For each condition 50 worms from 3 plates were counted and the experiment was repeated 3 times using independent batches of prepared extracts. Chi-squared test was used to assess statistical significance.

For induction assays with thalli and gun cells from strain NICo1, the extracts were made as described above and 100 µl was added to OP50 plates to fully cover the bacterial lawn. Approximately 400 C*. elegans* eggs were added to each plate. The plates were incubated at 20°C and scored for *chil-27p::GFP* induction after 48h using a Zeiss Axio Zoom V16 (Zeiss) microscope. Animals were classified in two groups (GFP positive and negative) and *col-12p::mCherry* expression was confirmed in both cases. For each condition, two independent extracts were prepared and applied onto separate plates. 30 worms were counted for each plate (n = 60). Chi-squared test was used to assess statistical significance.

For avoidance assays, either 10 infected worms or 100 µl of extract were added to 50 µl OP50 lawn on NGM plates as described above. 30 animals were added onto each plate with 5 plates per condition and the number of animals on the lawn was scored after 2h and 16h of incubation at 25°C. Chi-squared test was used to assess statistical significance.

### RNA-Sequencing

The experiment was conducted in biological triplicates. Synchronized L4 stage N2 animals were added onto NGM plates containing a 100 µl lawn of OP50 with multiple MBAo1 infected worms. The plates were incubated at 20°C and animals were collected by gentle washing with M9 buffer (Stiernagle, 2006) after 6 hours and 12 hours post-pathogen exposure. RNA was extracted using Trizol and quality was tested by agarose gel electrophoresis before sending for sequencing at BGI Genomics (Hong Kong). Pseudoalignment of raw reads was performed using Kallisto (Bray et al., 2016) and WS235 transcriptome from Wormbase to obtain the TPM (transcript per million) values for all genes. These counts were then analyzed by Sleuth with Wald test for two-sample comparisons to obtain a final list of differentially expressed genes (Pimentel et al., 2017). Overlaps between gene lists were assessed based on a hypergeometric test at nemates.org. The raw RNAseq data have been deposited to NCBI GEO under accession GSE175950.

### Gene Set Enrichment Analysis (GSEA)

The RNAseq datasets (6h and 12h exposure to *H. zoospora)* were compared to other *C. elegans* transcriptomic datasets using Gene Set Enrichment Analysis (GSEA) software v4.0.3 (Mootha et al., 2003; Subramanian et al., 2005). The genes that were significantly differentially expressed post pathogen exposure (FDR<0.1) were ranked based on their *b* value in descending order. We used 65 gene sets for the analysis derived from (Osman et al., 2018; Reddy et al., 2019) and WormExp [(Yang et al., 2016), which can be found in **Table S2**]. Pre-ranked analysis with weighted enrichment statistic, 1000 permutations and a minimum of 15 genes overlap, was performed independently for 6h and 12h post-pathogen exposure. The NES-values of gene sets with FDR<0.25 and nominal *p* value<0.05 were considered as significant and the results are summarized in **Table S3**.

### Transmission Electron Microscopy (TEM)

Samples for TEM were prepared following the conventional two-step fixation for TEM protocol 8 published in WormBook (Shaham, 2006) with few amendments. Briefly, N2 animals were exposed to pathogen for 6, 24 and 48 hours and controls were washed with M9 buffer, fixed for 1 hour at room temperature (2.5% glutaraldehyde, 1% paraformaldehyde in 0.1M sucrose, 0.05M cacodylate), rinsed and placed onto a glass slide to be cut in half, and placed back into fixing solution overnight at 4°C. Samples were washed 3x with 0.1M cacodylate, fixed in 0.5% OsO_4_, 0.5% K_4_Fe(CN)_6_ in 0.1M cacodylate for 90 minutes at 4 °C, followed by a 3x wash with 0.1M cacodylate. About 3-4 parallel aligned fixed worm halves were embedded close together in 2% low melt temperature agarose. Small agarose blocks containing the worm halves were cut out and dehydrated through an ethanol series at room temperature of 10 min 25% ethanol, 10 min 50% ethanol (or alternatively stored in 50% o/n at 4°C), 10 min 70% ethanol, 10 min 90% ethanol, 5x 8min 100% ethanol and at last 3x 10 min epoxypropane. Samples were infiltrated with resin (10g Agar 100, 8g DDSA, 5g NMA) through a series of 40min in 1:2 resin/epoxypropane, 40 min in 2:1 resin/epoxypropane, and 2h in absolute resin. Resin was taken off, the samples placed at 40°C for 10min to evaporate the remaining epoxypropane and placed back into fresh resin over night at room temperature. Samples were then placed into an embedding mold, covered with fresh resin and cured at 60°C for 2-3 days. 70 nm thick sections were cut and imaged using a JEOL JEM1010 TEM with a Gatan Orius camera.

### Scanning Electron Microscopy (SEM)

To perform scanning electron microscopy, mixed-stage populations were collected from plates containing the pathogen, washed twice with M9 and fixed for 3 hours at room temperature in a solution containing 3% glutaraldehyde (Sigma) in M9. Fixed animals were then washed twice in M9 and dehydrated gradually from 15% to 100% ethanol. Samples were dried in a critical point dryer (K850, ProSciTech) and coated with gold/palladium for 90s using the SC7620 Mini Sputter Coater (Quorum technologies). The samples were imaged in a JEOL JSM-6390 scanning electron microscope using 5 to 25 kVolt acceleration voltage.

## Results

### The Oomycete *Haptoglossa zoospora* Is a Potent Pathogen of *C. elegans*

**Figure 1:**
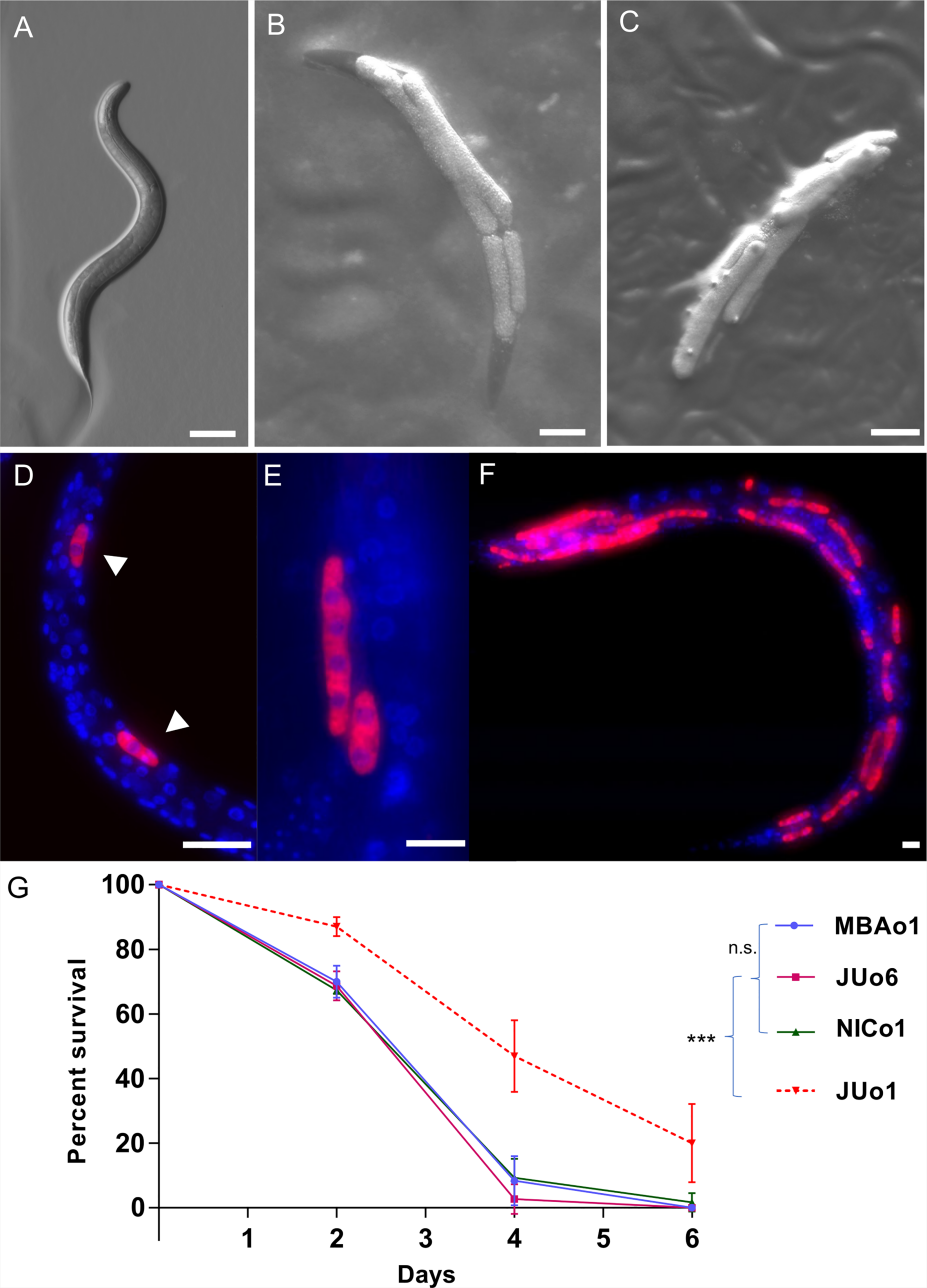
*H. zoospora* is a potent pathogen of *C. elegans*. **(A)** Uninfected wild-type *C elegans*. **(B, C)** Infected animals showing initially smooth thalli **(B)** and then thalli with exit tubes **(C)**. **(D–F)** Oomycete labelling by FISH shows the infection sites (arrowheads) across the body of the nematode and how these elongate to form the characteristic thalli. Red corresponds to pathogen 18S rRNA and blue to pathogen and *C. elegans* nuclei stained by DAPI. **(G)** Survival curve of *C. elegans* following exposure to the *M. humicola* strain JUo1 and *Haptoglossa* strains MBAo1, JUo6, and NICo1 at 25°C (***p < 0.001 based on log-rank test, n=90 worms per condition). Scale bars in **(A–C)** are 100 µm and in **(D–F)** are 10 µm.

To discover new oomycete pathogens infecting *C. elegans*, we turned to a site in Bedfordshire in England, where *Haptoglossa* infections had been previously observed. *Haptoglossa* is a basal group of oomycetes that diverged before the Saprolegnian and Peronosporalean clades (Hakariya et al., 2007; Hakariya et al., 2009; Beakes et al., 2012; Spies et al., 2016; Grover and Barkoulas, 2021). We used *C. elegans* N2 animals as a pathogen trap and infected worms were identified by the presence of sausage-like thalli, which appeared initially smooth (**Figures 1A, B**) and later developed short exit tubes to release infectious spores (**Figure 1C**). To identify the pathogen we used 18S and cytochrome oxidase 2 (*cox-2*) amplicons as barcodes (Choi et al., 2015). Sequencing of 18S amplicons revealed a sequence identity to *Haptoglossa zoospora* strains Y11 and LEV6507 (Spies et al., 2016). This was further supported by *cox-2* sequencing, which revealed high similarity to *Haptoglossa zoospora* strains (**Figure S1**). To consolidate the identification, we used fluorescence *in situ* hybridisation (FISH) targeting the 18S rRNA of *H. zoospora*. We detected the oomycete as single-cell sporidia inside the nematode body (**Figure 1D**), which developed to thalli that covered the entire length of the animal (**Figures 1E, F**). While the exact dynamics of this infection were dependent upon the availability of the pathogen and the likelihood of a nematode encountering a gun cell, the infection proceeded quickly with dead animals appearing within 24-48 hours post exposure to the pathogen. Moreover, in contrast to *M. humicola* (strain JUo1), this pathogen was extremely potent and all nematodes died upon pathogen exposure, therefore *Haptoglossa* cultures could only be maintained by introducing new nematodes (**Figures 1G**, **S2**). With regard to host specificity, *H. zoospora* successfully infected all *Caenorhabditis* species and other genera we tried such as *Pristionchus*, *Oscheius* and *Mesorhabditis* (**Figure S3**).

**Figure 2:**
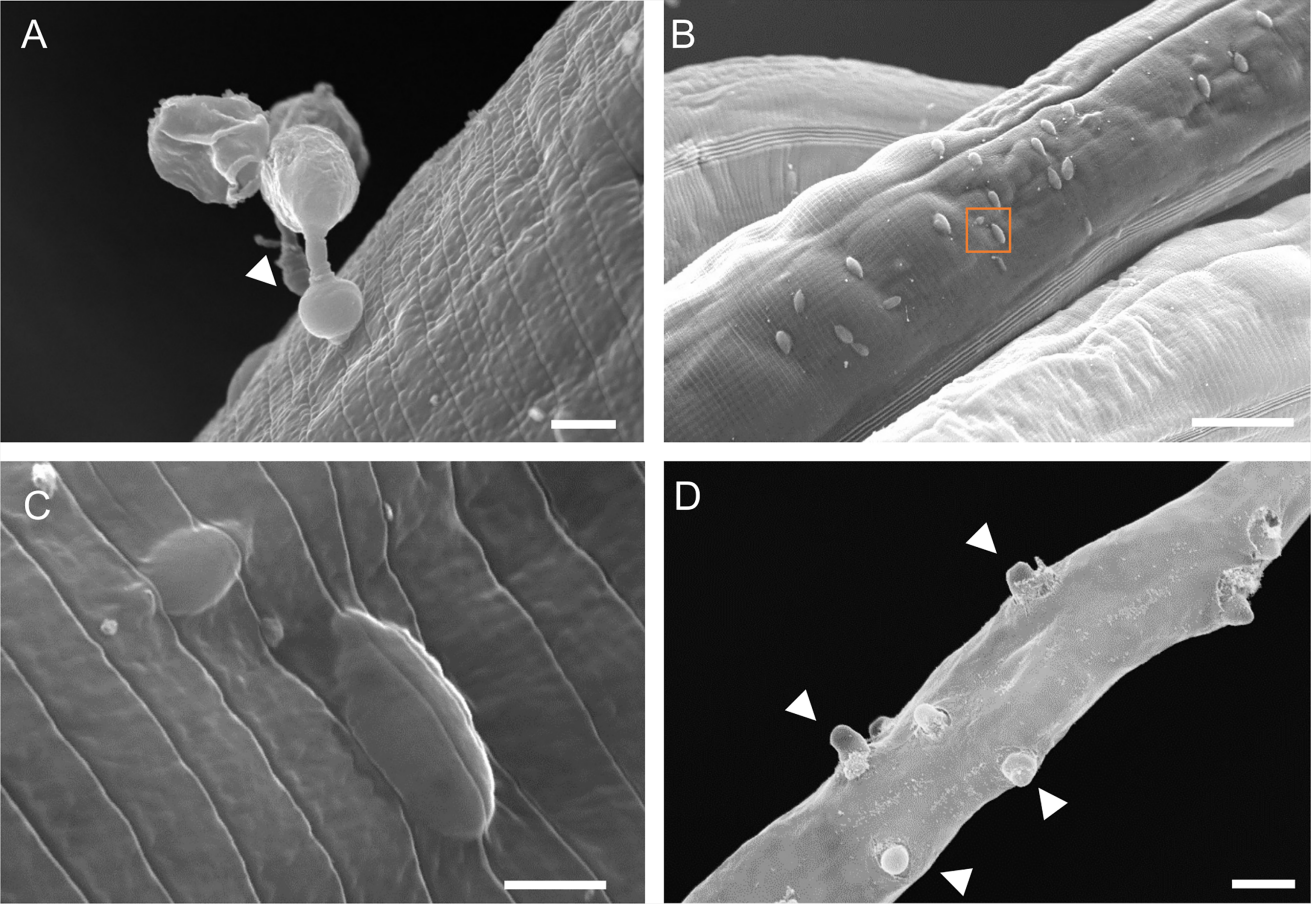
Scanning electron microscopy of *C. elegans* infection by *H. zoospora*. **(A)** Gun cell attached to the host cuticle. Arrowhead points to the inflated adhesorium. **(B)** Protrusions underneath the cuticle surface which correspond to injected sporidia and indicate multiple sites of infection 6 hours post pathogen exposure. **(C)** Zoomed image of orange box in B showing a sporidium underneath the cuticle. Note that there is no noticeable damage in the cuticle post injection. **(D)** Exit tubes develop through the cuticle to allow release of gun cells 24-48 hours post pathogen exposure. Arrowheads point to some exit tubes. Scale bars in **(A, C)** are 2 µm and in **(B, D)** are 20 µm.

Oomycetes of the *Haptoglossa* genus are known for their harpoon-like attack to infect nematodes (Barron, 1980; Robb and Barron, 1982; Beakes and Glockling, 1998; Beakes and Glockling, 2000; Glockling and Beakes, 2000a). This consists of firing a needle-like structure from a specialised gun cell to insert a single-cell sporidium inside the host body cavity (Hakariya et al., 2002). We noticed that when nematodes were added to plates containing *Haptoglossa*, the animals retracted upon encountering oomycete spores, suggestive of such “firing” events (**Video S1**). To visualise pathogen penetration, we performed scanning electron microscopy. We were able to observe gun cells attached to the host cuticle with an inflated adhesorium while injecting the sporidium into the *C. elegans* body (**Figure 2A**). These injection events led to multiple protrusions underneath the surface of the cuticle (**Figure 2B**), which likely correspond to the sporidia that we observed by FISH. It is of note that this injection process was found to be seamless, leaving almost no trace of damaged cuticle around the injected sporidia, and occurred on all regions of the cuticle (**Figures 2B, C**). At the late stages of infection, the thalli formed short exit tubes (**Figure 2D**) that disrupted the integrity of the cuticle by projecting through it to allow release of the gun cells.

### Characterisation of the Growth of *H. zoospora* Inside the Body of *C. elegans*

**Figure 3:**
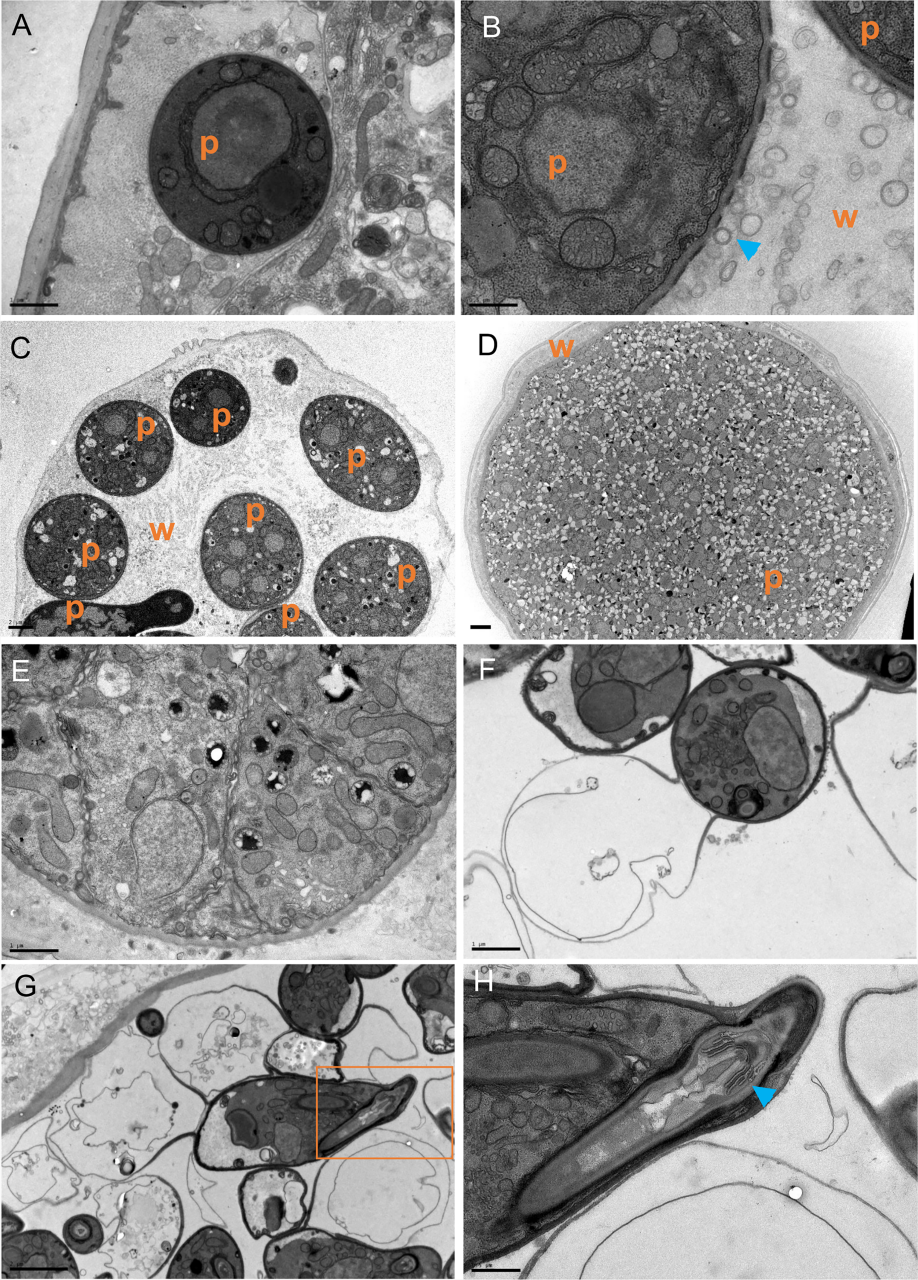
The growth and development of *H. zoospora* inside *C. elegans*. **(A)** The sporidia are initially found intracellularly just below the cuticle 6 hours post exposure. **(B)** Within 24 hours, extensive host tissue degradation is observed possibly through the secretion of degrading enzymes *via* vesicles (blue arrowheads) seen near gaps of the oomycete cell wall. **(C, D)** The thalli continue to grow until they have filled the body of the host while the cuticle remains intact. Note several thalli in **(C)** and one big thallus covering the entire width of the animal in **(D)**. In panels **(A–D)**, “p” indicates the position of pathogen and “w” marks the worm tissues. **(E)** Thalli become compartmentalized to form zoospores and transition to gun cells. **(F)** Zoospores undergo encystation to form gun cells. **(G)** A gun cell is shown with a vacuole on end and the injection apparatus on the other within the orange box. **(H)** Zooming into the orange box in G to show the gun cell detail with the needle (blue arrowhead) and bore. Scale bars are 0.5 μm in **(B, H)**; 1μm in **(A, F)**; 2μm in **(C, D, G)**.

To understand how the oomycete grows within the body of *C. elegans* we performed transmission electron microscopy (TEM) during the infection process. Cross-sections at early stages of infection (6 hours post-exposure) revealed that *H. zoospora* sporidia were first positioned intracellularly within muscles or the epidermis (**Figure 3A**) before they started to progressively disrupt the tissues of the host. This disruption was evident already within 24 hours post pathogen exposure, and based on our observation of pores on the pathogen cell wall (**Figure 3B**), it may rely on active secretion of degrading enzymes. This process eventually led to complete digestion of host tissues within 48 hours, with one or more thalli filling up the entire body cavity depending on the initial number of sporidia that initiated the infection (**Figures 3C, D**). Interestingly, the pathogen thalli continued to be protected by an intact cuticle until host tissue digestion was completed (**Figures 3C, D**). The multi-nucleated thalli became compartmentalised to form zoospores, which underwent encystation to form the infectious gun-cells (**Figures 3E, F**). The gun cell consisted of a vacuolated base used to push the protoplasmic mass of the sporidium inside the host through a specialised needle located at the tip of the gun cell (**Figures 3G, H**). The observed gun cell and sporidium morphology are consistent with previous *H. zoospora* descriptions (Hakariya et al., 2002) and highlight that the oomycete can grow within the body cavity of *C. elegans* by degrading all internal host tissues.

### Infection by *H. zoospora* Induces a Transcriptional Response in *C. elegans* That Shares Features With the Response to *M. humicola*

**Figure 4:**
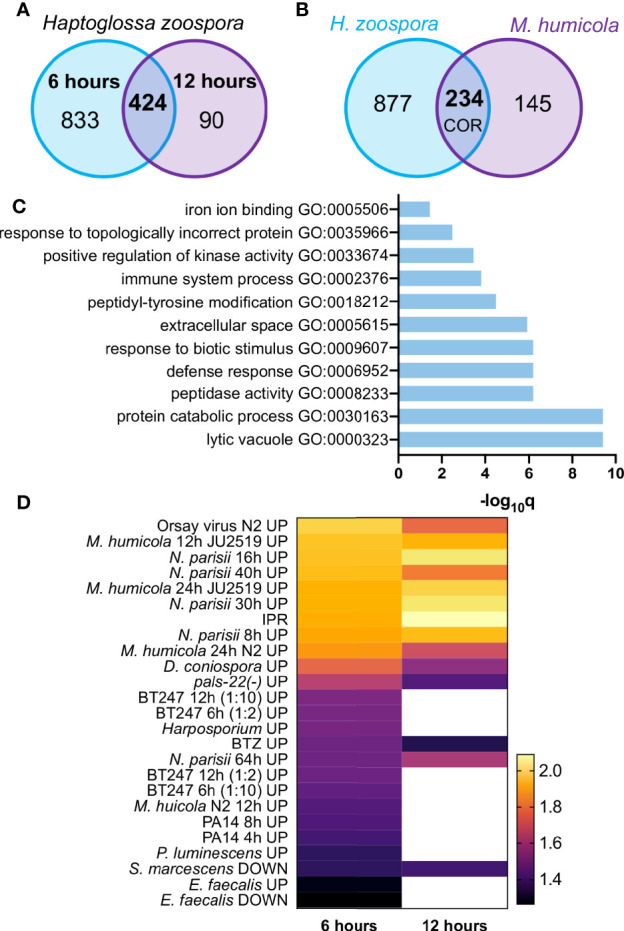
The transcriptional response of *C. elegans* to *H. zoospora* infection. **(A)** Venn diagram showing numbers and overlap of up-regulated genes 6 h and 12 h post infection with *H. zoospora* (hypergeometric test: Representation Factor RF: 4.1, p < 0.0001). **(B)** Venn diagram showing overlap of up-regulated genes between *H. zoospora* and *M. humicola* infection, corresponding to the ‘common oomycete response (COR)’ genes (hypergeometric test: RF: 4.3, p < 0.0001). **(C)** GO term analysis for COR genes identifies terms related to biotic stress. **(D)** Heatmap presenting the normalised enrichment score (NES) by GSEA analysis for gene sets showing significant intersection with *H. zoospora* infection datasets. White colour depicts no significant intersection (FDR < 0.25 and nominal *p* value < 0.05).

To study how *C. elegans* responds to *Haptoglossa* infection, we performed RNA-seq during the course of infection. To this end, L4 stage N2 animals were exposed to *H. zoospora* on NGM plates that contained a dense population of gun cells to maximise the possibility that nematodes would come in contact with the pathogen. Since animals die fast from this infection, we chose to analyse two early time points, 6 and 12 hours after exposure to the pathogen. These represent stages where infected animals only display early symptoms, such as difficulty in locomotion, without having visible thalli within their body. Our analysis revealed 1257 genes significantly upregulated at the 6-hour time point and 514 genes at the 12-hour time point (**Figure 4A** and **Table S1**). Out of these, 424 genes were found to be shared between the two timepoints (**Figure 4A**). We reasoned that genes differentially expressed upon *H. zoospora* exposure may be shared with those responding to *M. humicola* infection, thereby representing a common oomycete response (COR). Indeed, we found 235 upregulated genes that were in common between the *Haptoglossa* and *Myzocytiopsis* infection datasets (**Figure 4B** and **Table S2**). Gene ontology (GO) analysis (Angeles-Albores et al., 2016) of differentially expressed genes showed significant enrichment for terms related to infection, such as “response to biotic stimulus”, “defense response” and “immune system process” (**Figures 4C** and **S4**). Other highly enriched GO terms were related to signalling upon stress and catabolic processes (**Figures 4C** and **S4**).

Next, we compared the transcriptional response to *H. zoospora* with the response against other known pathogens of *C. elegans* using gene set enrichment analysis (GSEA) (Mootha et al., 2003; Subramanian et al., 2005). We found that *H. zoospora* exposure at 6 and 12 hours showed significant intersection (nominal [NOM] p value < 0.05 and false discovery rate [FDR] < 0.25) with 25 and 14 out of 50 datasets analysed respectively (**Table S2**). The most significant intersection was found with datasets derived from infection by *M. humicola* and *N. parisii* or the Orsay virus (**Figure 4D**, **Table S2**). This is not very surprising in light of the recently reported overlap between the response to *M. humicola* detection and the induction of IPR upon exposure to microsporidia or impairment of the negative regulator PALS-22 (Bakowski et al., 2014; Chen et al., 2017; Reddy et al., 2019; Fasseas et al., 2021). Some less significant overlap observed with other fungal and bacterial infection datasets (Troemel et al., 2006; Engelmann et al., 2011; Yang et al., 2015) may be attributable to response to generic tissue damage caused by the growth of *Haptoglossa* inside the nematode (**Figure 4D**).

**Figure 5:**
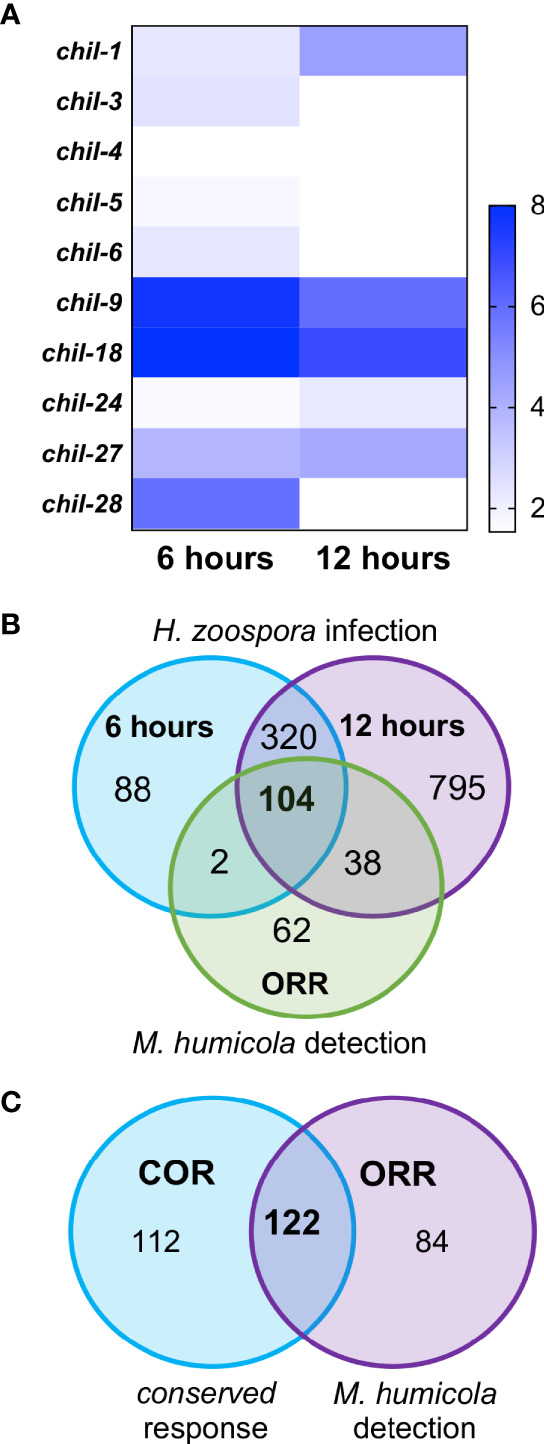
Conservation between *C. elegans* response to *H. zoospora* infection and *M. humicola* recognition. **(A)** Heatmap showing the members of the *chil* gene family that are differentially expressed upon *H. zoospora* infection. Heatmap reflects Sleuth b values, which are analogous to fold change. Absence of any value indicates that the gene was not significantly up-regulated (*p* value < 0.01 and FDR adjusted *p* value < 0.1) in that condition. **(B)** Venn comparison of the transcriptional response *H. zoospora* infection with the oomycete recognition response previously reported for *M. humicola*. Note that the majority of ORR genes overlap with genes induced upon *H. zoospora* infection. **(C)** Venn comparison between the conserved oomycete response (COR) and oomycete recognition response (ORR). Again the majority of ORR or COR genes are shared (hypergeometric test: RF: 38.6, p < 0.0001).

We previously reported that a hallmark of the *C. elegans* response to *M. humicola* infection is the induction of *chil* genes, which occurs upon detection of *M. humicola* without infection (Osman et al., 2018; Fasseas et al., 2021). Among the identified COR genes, the *chil* gene family was also well represented (**Figure 5A**), pointing to similarities in the host response against oomycetes that are phylogenetically distinct and exhibit distinct infection strategies. Intrigued by this finding, we sought to determine the overlap between the response to *H. zoospora* infection and the oomycete recognition response (ORR) recently described upon exposure to an extract prepared from animals infected by *M. humicola* (Fasseas et al., 2021). We found that more than 50% of ORR genes overlapped with the *H. zoospora* infection datasets (**Figure 5B**) and COR genes (**Figure 5C** and **Table S2**). Taken together, we conclude that the response to *H. zoospora* infection shares similarities with the response against *M. humicola* infection, and that a large part of this similarity reflects genes that are likely to be induced upon pathogen detection.

### *C. elegans* Can Detect *Haptoglossa* Oomycetes Without Infection

**Figure 6:**
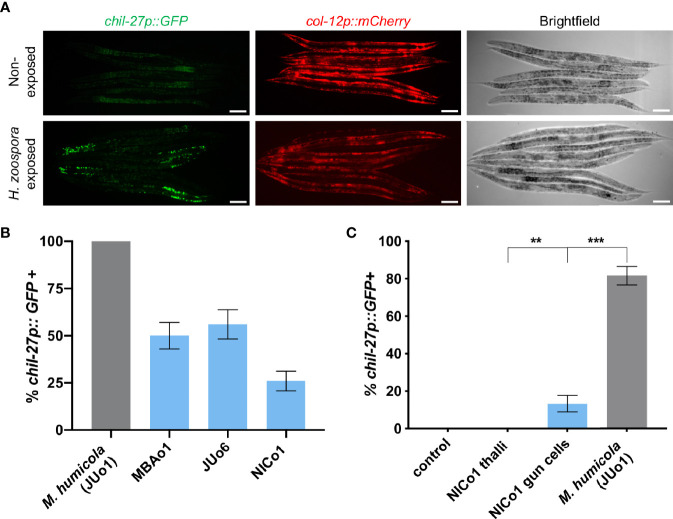
*C. elegans* can detect *Haptoglossa* oomycetes without infection **(A)** Induction of *chil-27p::GFP* in worms on a *H. zoospora* infection plate. Scale bar, 40 μm. **(B)** Induction assay comparing *chil-27p::GFP* induction in response to extracts derived from *M. humicola* and *Haptoglossa*. Our *Haptoglossa* extracts are consistently less potent than *M. humicola* although we found that they all induce variably the same response. **(C)** Induction assay comparing *chil-27p::GFP* induction in response to control extract made from N2 adults, NICo1 thalli extract, NICo1 gun cell extract, and *M. humicola* extract. NICo1 gun cell extract induces *chil-27p::GFP*, albeit at a significantly lower extent compared to *M. humicola* extract. NICo1 thalli extract failed to induce *chil-27p::GFP* (^∗∗∗^p value < 0.001 and ^∗∗^p value < 0.01 with a chi-squared test, n = 60 for each extract treated group).

The induction of *chil* genes has been previously shown to be independent of *M. humicola* penetration (Osman et al., 2018). Therefore, the induction of *chil* genes and overall similarity in the transcriptional response raised the possibility that *C. elegans* can also recognize *H. zoospora* in the absence of any infection. We first exposed animals carrying the *chil-27p::GFP* transgene to *H. zoospora* to validate that the marker can be rapidly induced, as shown in the case of *M. humicola*. After 24 hours of exposure, GFP signal was observed in most animals on the plate (**Figure 6A**). To test if the response is associated with pathogen detection, we treated animals with pathogen extracts (see methods) in comparison to control extracts made from non-infected animals. We found that exposure to filtered pathogen extracts resulted in up to 50% activation of *chil-27p::GFP* in the population, although these animals did not show any sign of infection even several days post exposure. Furthermore, no avoidance behaviour was found in animals after exposure to the pathogen or *Haptoglossa*-derived extract (**Figure S5**). These results suggest that the induction of *chil-27* is not because of *Haptoglossa* infection and is likely to be mediated by pathogen detection.

Over the course of these experiments, we identified additional *Haptoglossa* strains in Japan (JUo6) and Taiwan (NICo1) naturally infecting *Caenorhabditis* and *Oscheius* nematodes respectively. These strains infect *C. elegans* with the same efficacy as *H. zoospora* MBAo1 (**Figure 1G**) and have similar infection strategy (**Figure S6**). Extracts prepared from these oomycetes also induced *chil-27p::GFP* expression (**Figure 6B**). Instead of motile zoospores, JUo6 and NICo1 produce aplanospores, which develop into larger gun cells that concentrate around their point of release (Hakariya et al., 2002; Hakariya et al., 2007; Hakariya et al., 2009) (**Figure S2**). This mode of release allowed us to test whether gun cells were able to induce *chil* gene expression. Remarkably, extracts prepared by heat-inactivating NICo1 gun cells were sufficient in inducing *chil-27p::GFP* expression without causing any symptoms of infection (**Figure 6C**). In comparison, extracts prepared from NICo1 thalli were less efficient in inducing *chil-27p::GFP* expression. Taken together, we conclude that *chil* gene induction is a general response to detection of different nematode-infecting oomycetes and may rely on host detection of a molecule that is more abundant in the infectious gun cells.

## Discussion

Our study expands the repertoire of known natural pathogens of *Caenorhabditis* nematodes (Schulenburg and Felix, 2017) by introducing *Haptoglossa* species. Together with *Myzocytiopsis*, this is the second oomycete genus now reported to infect *C. elegans.* The morphological description of the *Haptoglossa* infection highlights that these pathogens reach the body cavity by going through the first outer cell layer and then growing in the body cavity by degrading internal host tissues, with the exception of the cuticle that remains intact and encapsulates the developing thalli until the very late stages of infection. Nevertheless, *Haptoglossa* infections show some key differences compared to those by *M. humicola.* First, *Haptoglossa* species infect *C. elegans* more efficiently than *M. humicola*, which never eradicates the population. Secondly, *Haptoglossa* relies on specialized cells, the gun cells, to inject a single cell sporidium within the host. This injection apparatus is reminiscent of the microsporidian polar tube, which everts to pierce the membrane of the target host cell (Troemel et al., 2008; Jaroenlak et al., 2020). Thirdly, in contrast to *M. humicola* that preferentially attaches to the mouth and alae of the animal (Osman et al., 2018), *H. zoospora* attaches to all regions of the cuticle. Furthermore, the *Haptoglossa* thalli do not bifurcate and instead grow along the longitudinal axis of the animal to give rise to sausage-like thalli, as opposed to the “pearls” previously reported for *M. humicola* sporangia (Osman et al., 2018).

Despite these differences in the oomycete infection strategies, similarities were revealed with regard to the host response against *H. zoospora* and *M. humicola*, including the induction of the *chitinase-like* gene family (Osman et al., 2018). This finding emphasises the potential importance of CHIL-mediated defence as a means for nematodes to prepare for an oomycete attack (Osman et al., 2018). Interestingly, the induction of *chil* genes in both cases appears to be a consequence of pathogen detection as opposed to infection, because it can be triggered upon exposure to non-infectious extracts. Furthermore, most of the shared response to *M. humicola* and *Haptoglossa* (COR) overlapped with the previously characterized response to oomycete recognition (ORR). Based on these findings, we speculate that the same signalling pathway may lead to *chil* gene induction in the epidermis following oomycete detection in neurons *via* cross-tissue communication (Fasseas et al., 2021). This scenario is consistent with growing evidence on neuronal regulation of various immune defenses in *C. elegans* (Hoffman and Aballay, 2019; Wani et al., 2020). The fact that *C. elegans* can recognize all tested *Haptoglossa* strains further strengthens that oomycete-nematode interactions are likely to be frequent in the wild. Considering the origin of *Haptoglossa* strains collected from diverse locations around the world and the fact that *Myzocytiopsis* and *Haptoglossa* are phylogenetically distinct pathogens (Beakes et al., 2012; Spies et al., 2016), we propose that the induction of *chil* genes may be a universal *C. elegans* response to an oomycete threat.

*C. elegans* has been shown to develop strong aversive behavior towards several pathogens (Kim and Flavell, 2020; Singh and Aballay, 2020), however, we did not observe avoidance behavior towards either the oomycete pathogens or pathogen-derived extracts. This suggests that pathogen detection in this case is more likely to trigger a “fight” rather than “flight” response. It is also possible that interactions with other microbes in the wild can modulate avoidance towards oomycete pathogens, and this idea can be tested in the future using the recently described natural microbiome of *C. elegans* (Dirksen et al., 2020).

While *chil* gene induction upon pathogen detection may be a common feature between *Haptoglossa* and *Myzocytiopsis* recognition, the response is likely to be triggered by a different molecule in each case. Despite the fact that *Haptloglossa* infections are easier to propagate in nematode populations, we found that the potency of *Haptoglossa* extracts to induce *chil-27::GFP* was consistently lower than those prepared from *M. humicola.* This suggests that a distinct oomycete molecular pattern may be present in the unique infectious cells of *Haptoglossa*, which are missing in *M. humicola*. Extracts from both oomycetes will be subjected in the future to metabolomics analysis to identify their active constituents. Furthermore, the establishment of *C. elegans* as a model will allow genetic work to dissect the signalling pathway including the host receptors involved in oomycete perception.

The identity of wild nematodes found infected by *Haptoglossa* has rarely been assessed (Glockling and Beakes, 2002), but a broad host range was expected given that these oomycetes have been reported to also infect rotifers (Barron, 1980; Robb and Barron, 1982). We found that all nematodes we tested were susceptible to infection, including *Pristionchus pacificus* which was previously found to be resistant to *M. humicola* (Osman et al., 2018). Nematode-killing pathogens have been proposed as biocontrol agents for animal and plant parasitic nematodes to protect livestock and minimise losses in economically important crops (Nordbring-Hertz et al., 2011). Oomycetes, as broad range nematode pathogens, may offer new opportunities for biocontrol and our ability to grow them in the lab using *C. elegans* could streamline the production of such biocontrol agents.

## Data Availability Statement

The datasets presented in this study can be found in online repositories. The names of the repository/repositories and accession number(s) can be found in the article/**Supplementary Material**.

## Author Contributions

MG and MF carried out the majority of the experiments. CE performed the TEM microscopy. KL contributed transgene induction assays. CB, M-AF, and SG sampled the new pathogen strains. MB supervised and led the work. All authors contributed to writing and editing of the manuscript. All authors contributed to the article and approved the submitted version

## Funding

We would like to thank the Wellcome Trust [219448/Z/19/Z] for supporting this work.

## Conflict of Interest

The authors declare that the research was conducted in the absence of any commercial or financial relationships that could be construed as a potential conflict of interest.

## Publisher’s Note

All claims expressed in this article are solely those of the authors and do not necessarily represent those of their affiliated organizations, or those of the publisher, the editors and the reviewers. Any product that may be evaluated in this article, or claim that may be made by its manufacturer, is not guaranteed or endorsed by the publisher.

## Supplementary Material

Supplementary Figure 1 Phylogenetic analysis of *Haptoglossa* strains. The phylogenetic tree was inferred by Maximum Likelihood based on 14 *cox2* sequences using MEGA X (Kumar et al., 2018). The tree with the highest log likelihood is shown and is drawn to scale with branch lengths measured in the number of substitutions per site. The new *Haptoglossa* isolates are highlighted in blue and *M. humicola* (JUo1) was used as an outgroup. The following 10 sequences with Genbank accession number were used: AB437406 SZ03; AB437405 Y11; AB253786 SZ01; KT257467 LEV6507; AB253780 TK01; AB253781 NA01; AB253782 TK02; AB253783 TK03; AB253785 NI01; AB253784 KG01.

Supplementary Figure 2 Infection phenotypes caused by *Haptoglossa* strains. *Haptoglossa* sp. are highly potent pathogens, with all nematodes dying from their attack under lab conditions. **(A)** *C. elegans* infected by MBAo1. **(B)** Zoomed image of MBAo1 infected animals showing sausage-like thalli and multiple exit sites for release of zoospores. **(C)** *C. elegans* infected by NICo1 showing presence of clusters of spores throughout the plate. **(D)** Zoomed image of NICo1 infected animals showing spore clusters around the bodies of dead worms. **(E, F)** Single animals are shown of Juo6 infection **(E)** and MBAo1 infection **(F)** to highlight how spores are clustered around dead worms in infections by JUo6. **(G, H)** Gun cells produced by *Haptoglossa* isolates. **(G)** NICo1 produces larger gun cells compared to MBAo1 **(H)**. Scale bar is 500 µm in **(A, C)**; 100 µm in **(B, D–F)**; 10µm in **(G, H)**.

Supplementary Figure 3
*H. zoospora* is an oomycete with a broad host range. Susceptibility to infection by *H. zoospora* analyzed for other isolates of *C. elegans*, other *Caenorhabditis* species and other nematodes. Unlike *M. humicola*, *H. zoospora* could infect all tested nematodes.

Supplementary Figure 4 Gene ontology enrichment analysis of up-regulated genes identified at different timepoints. Two time points are shown 6 hours **(A)** and 12 hours **(B)** post *H. zoospora* exposure. All datasets have been analysed using the enrichment analysis tool on Wormbase.

Supplementary Figure 5
*C. elegans* does not avoid *Haptoglossa zoospora* or extracts derived from it. Avoidance assay showing percentage of animals leaving the lawn 2h and 16h post exposure to *H. zoospora* or pathogen extract at 25°C. Five plates with 30 worms on each were used for every condition (n=150). Ten dead worms were added on to each OP50+MBAo1 plates thereby maintaining a ratio of 1:3 for dead:alive animals. No significant difference was observed between the lawn distribution of pathogen-exposed or extract-exposed vs unexposed worms with a Chi-squared test.

Supplementary Figure 6 Growth of the NICo1 strain of *Haptoglossa* inside *C. elegans.* **(A, B)** Pathogen by FISH shows single sporidia at infection sites **(A)** and developing thalli **(B)**. Red corresponds to pathogen 18S rRNA and blue to *C. elegans* and pathogen nuclei stained by DAPI. **(C–H)** TEM images of the infection process starting from single sporidia within 6 hours (C), which grow into thali and cause tissue degradation within 24-48 hours **(D–F)**. In panels **(C–H)**, “p” marks pathogen and “w” worm tissues. **(G, H)** Compartmentalisation of thalli and aplanospore spore formation at the 48 hour time point. Scale bars are 2 µm in **(C, D, F–H)** and 10 µm in **(A, B, E)**.

Supplementary Table 1 RNA-seq results of *C. elegans* 6 hour and 12 hour post *H. zoospora* exposure. Sheet 1 gives the summary of total differentially expressed genes. Sheet 2 (6 h) and Sheet 3 (12 h) give the list of all differentially expressed genes along with their transcript names, Wormbase Gene IDs, gene/sequence names, Wald test p value and p-value adjusted for multiple test correction.

Supplementary Table 2 Gene lists of COR genes and overlap between COR and ORR genes.

Supplementary Table 3 GSEA analysis of genes upregulated upon *H. zoospora* exposure. Gene sets showing significant intersection with up-regulated genes at 6 h (Sheet 1) and 12 h (Sheet 2) post MBAo1 exposure (NOM p-value<0.05, FDR q-value<0.25 are considered significant). Sheet 3 gives the list of gene sets used in GSEA analysis. Gene sets with overlap > 25 genes were included in the analysis.

Supplementary Video 1 N2 animal retracting upon contact with *Haptoglossa sp.* NICo1 spores/gun cells.
