# Supplementary material for: Infection of C. elegans by Haptoglossa Species Reveals Shared Features in the Host Response to Oomycete Detection
Source: Front Cell Infect Microbiol. 2021 Oct 14;11:733094. doi: 10.3389/fcimb.2021.733094 (PMC8552708; doi:10.3389/fcimb.2021.733094)
Supplement: Supplementary Figure 1 — Phylogenetic analysis of Haptoglossa strains. The phylogenetic tree was inferred by Maximum Likelihood based on 14 cox2 sequences using MEGA X (Kumar et al., 2018). The tree with the highest log likelihood is shown and is drawn to scale with branch lengths measured in the number of substitutions per site. The new Haptoglossa isolates are highlighted in blue and M. humicola (JUo1) was used as an outgroup. The following 10 sequences with Genbank accession number were used: AB437406 SZ03; AB437405 Y11; AB253786 SZ01; KT257467 LEV6507; AB253780 TK01; AB253781 NA01; AB253782 TK02; AB253783 TK03; AB253785 NI01; AB253784 KG01. [file Image_1.pdf]

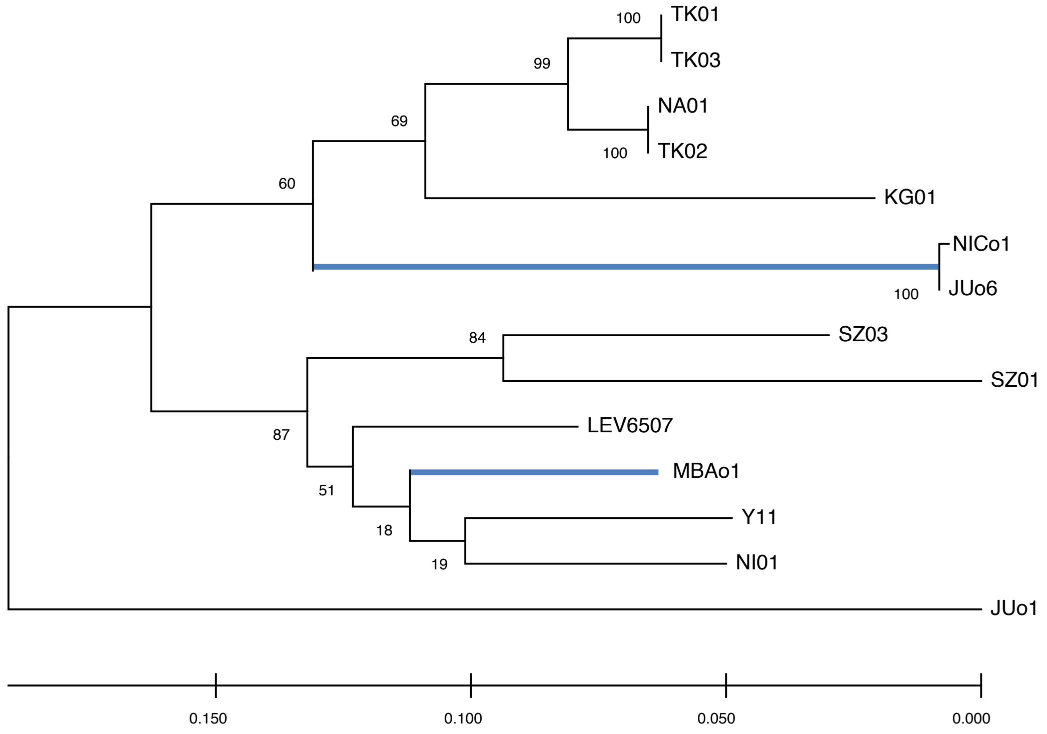
TK01
TK03
NA01
TK02
KG01
NICo1
JUo6
SZ03
SZ01
LEV6507
MBAo1
Y11
NI01
JUo1
100
99
69
100
60
100
84
87
51
18
19
0.150
0.100
0.050
0.000

Figure S1

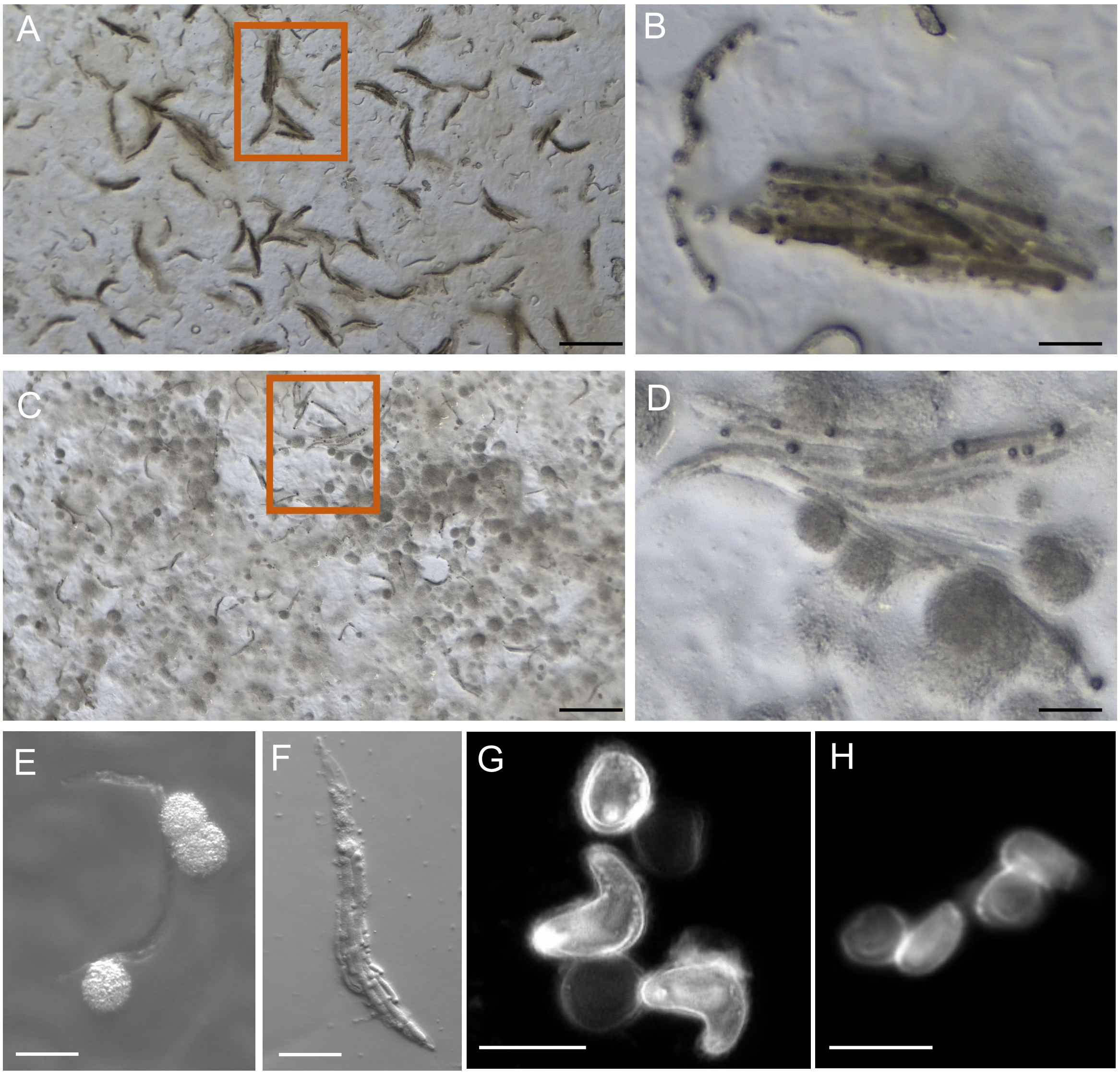

Figure S2

	**Infected by *H. zoospora* MBAo1**	**Infected by *M. humicola* JUo1**
C. elegans N2	Yes	Yes
C. elegans CB4856	Yes	Yes
C. elegans JU2519	Yes	Yes
C. briggsae AF16	Yes	Yes
C. remanei PB4641	Yes	Yes
C. angaria RGD1	Yes	Yes
O. tipulae CEW1	Yes	Yes
P. pacificus PS312	Yes	No
M. spiculigera MBA1180	Yes	ND

Figure S3

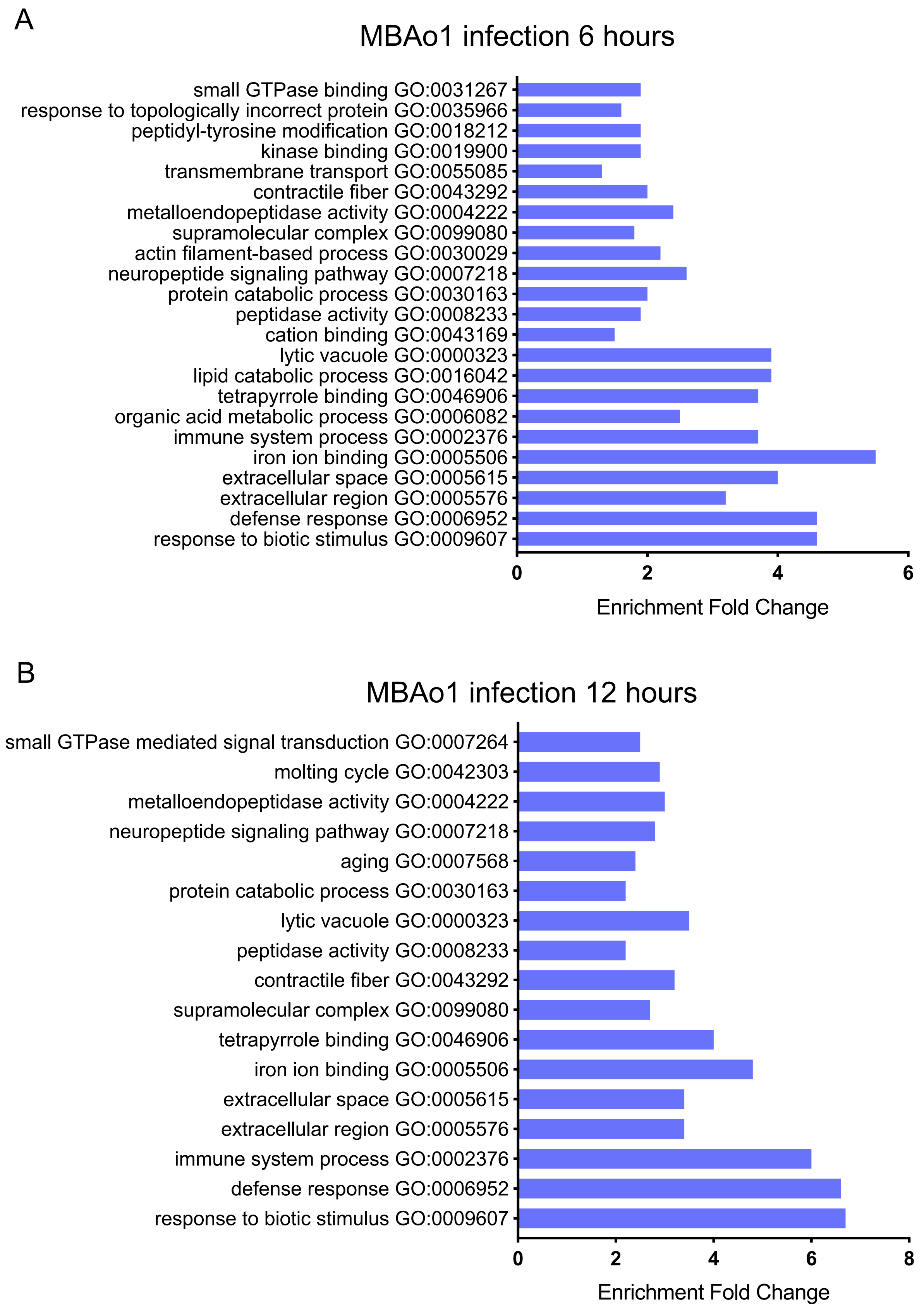
A
MBAo1 infection 6 hours
small GTPase binding GO:0031267
response to topologically incorrect protein GO:0035966
peptidyl-tyrosine modification GO:0018212
kinase binding GO:0019900
transmembrane transport GO:0055085
contractile fiber GO:0043292
metalloendopeptidase activity GO:0004222
supramolecular complex GO:0099080
actin filament-based process GO:0030029
neuropeptide signaling pathway GO:0007218
protein catabolic process GO:0030163
peptidase activity GO:0008233
cation binding GO:0043169
lytic vacuole GO:0000323
lipid catabolic process GO:0016042
tetrapyrrole binding GO:0046906
organic acid metabolic process GO:0006082
immune system process GO:0002376
iron ion binding GO:0005506
extracellular space GO:0005615
extracellular region GO:0005576
defense response GO:0006952
response to biotic stimulus GO:0009607
0
2
4
6
Enrichment Fold Change
B
MBAo1 infection 12 hours
small GTPase mediated signal transduction GO:0007264
molting cycle GO:0042303
metalloendopeptidase activity GO:0004222
neuropeptide signaling pathway GO:0007218
aging GO:0007568
protein catabolic process GO:0030163
lytic vacuole GO:0000323
peptidase activity GO:0008233
contractile fiber GO:0043292
supramolecular complex GO:0099080
tetrapyrrole binding GO:0046906
iron ion binding GO:0005506
extracellular space GO:0005615
extracellular region GO:0005576
immune system process GO:0002376
defense response GO:0006952
response to biotic stimulus GO:0009607
0
2
4
6
8
Enrichment Fold Change

Figure S4

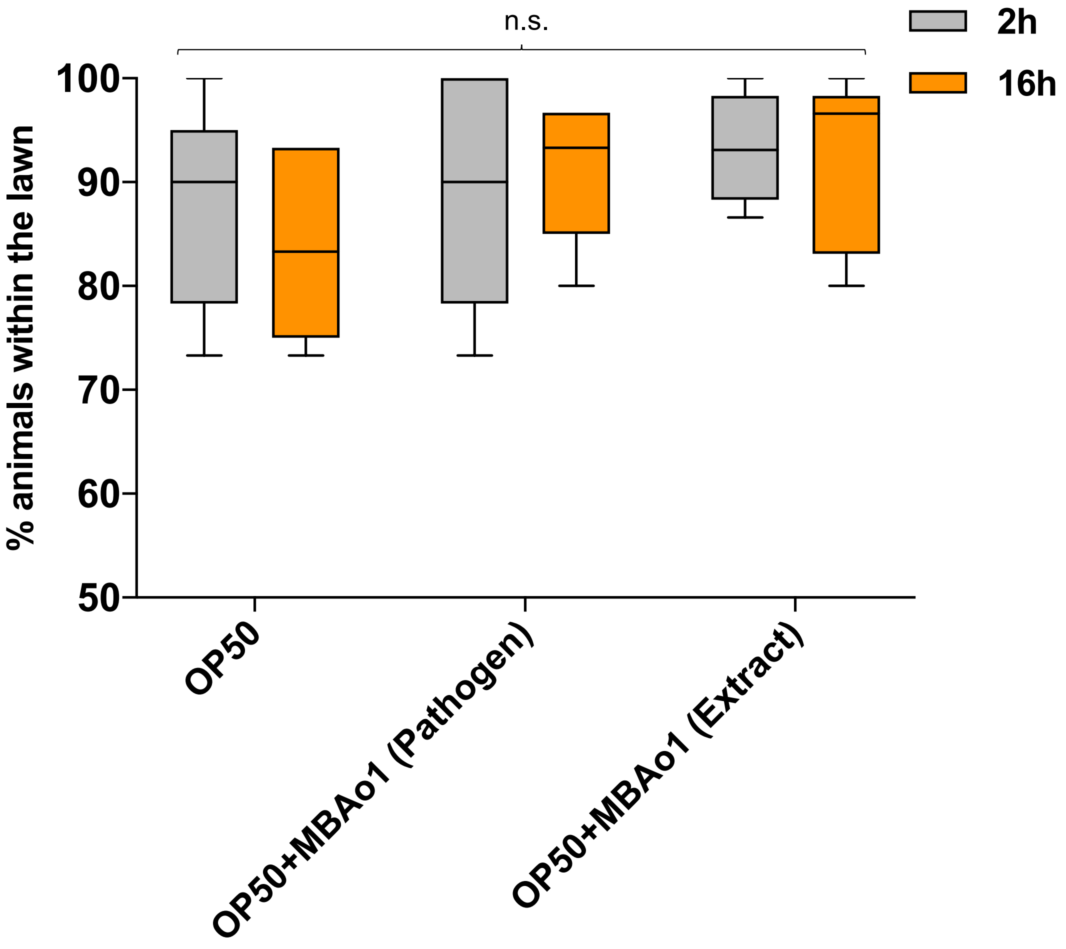
n.s.
2h
16h
% animals within the lawn
100
90
80
70
60
50
OP50
OP50+MBAo1 (Pathogen)
OP50+MBAo1 (Extract)

Figure S5

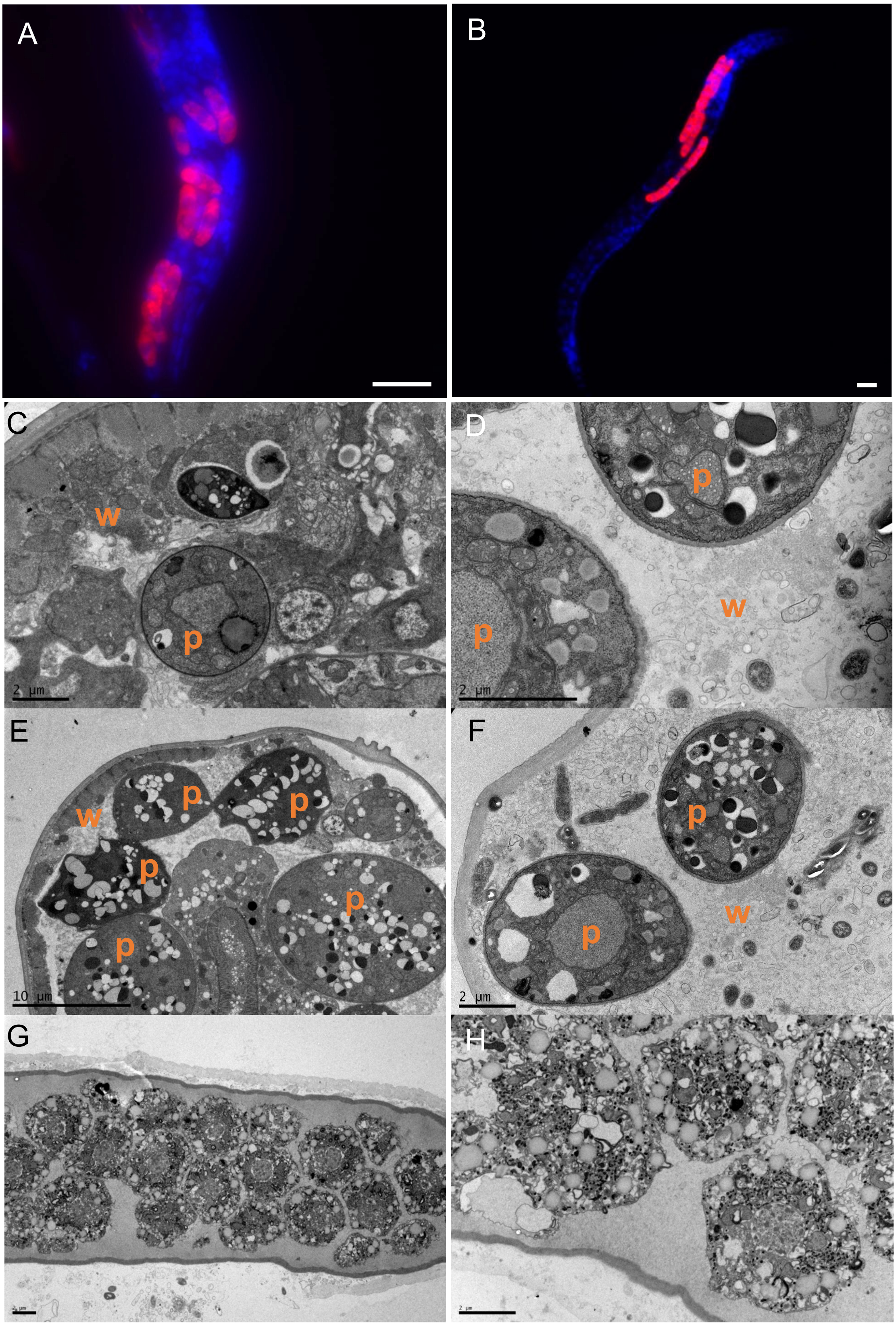

Figure S6
